# Healthcare providers’ educational role and Saudi pregnant women’s knowledge of active labor signs: a cross-sectional correlational study

**DOI:** 10.25122/jml-2025-0145

**Published:** 2025-12

**Authors:** Dalal Yahya Felemban, Abeer Saad Eswi, Hawazen Rawas

**Affiliations:** 1 King Abdullah International Medical Research Center, Jeddah, Saudi Arabia; 2 College of Nursing, King Saud Bin Abdulaziz University of Health Science, Jeddah, Saudi Arabia; 3 Ministry of the National Guard - Health Affairs; 4 Faculty of Nursing, Cairo University, Cairo, Egypt; 5 Nursing Department, College of Medicine and Health Sciences, Arabian Gulf University, Manama, Bahrain

**Keywords:** knowledge, active signs of labor, health care providers, educational role, pregnancy, Saudi women

## Abstract

Identifying the indicators of active labor is crucial for timely intervention, enhancing care focused on women, and minimizing unnecessary hospital admissions during the early stages of labor. This research aimed to evaluate the relationship between the educational role of healthcare providers and the knowledge of active labor signs among pregnant Saudi women. A descriptive cross-sectional correlational study was conducted at the Obstetrics and Gynecology Outpatient Department of King Abdulaziz Medical City in Jeddah. A convenience sample of 399 pregnant Saudi women completed a structured questionnaire. Over half of the participants (57.4%) were aged 26-35. Almost all respondents (97.7%) accurately identified cervical dilatation, and 95.5% recognized regular severe abdominal or back pain as signs of active labor. Family, friends, and online platforms were the most frequently cited sources of information (22.2% and 22.1%, respectively). The majority of participants (96%) felt comfortable inquiring about labor signs with healthcare providers, and 71.9% rated communication with providers as effective or very effective. Women showed a good understanding of active labor signs and felt comfortable communicating with healthcare providers. However, informal sources are still prevalent, underscoring the need for structured, provider-led education. It is recommended to incorporate targeted health education programs into routine antenatal visits to improve accurate knowledge and reduce reliance on non-professional sources

## INTRODUCTION

Childbirth is a pivotal life event signifying transition and new beginnings, with psychological, social, and emotional significance for the woman and family. Raising awareness about labor signs shows that positive birth experiences lead to better outcomes, while negative ones may cause postpartum depression and anxiety [1]. Healthcare providers’ (HCPs) support influences women’s agency before childbirth [2]. Research shows that childbirth classes positively impact pregnancy outcomes. Educational programs enhance pregnant women’s awareness of labor and prepare them for childbirth, focusing on the labor process, pain management, and emotional issues. This preparation reduces anxiety and increases confidence [3,4]. Understanding labor-onset signs is crucial for women-centered support and for improving early labor care [5]. Birth preparedness and complication readiness (BPCR) interventions can reduce maternal mortality through qualified birth attendants, healthcare facilities, transportation, blood donors, financial preparedness, and knowledge of danger signs [6]. Pregnancy and childbirth are physiological events with biological, psychological, and sociocultural aspects [7]. Labor onset marks the transition from pregnancy to birth [5]. For nulliparous women, lack of experience can increase doubt and anxiety [8], leading to unnecessary healthcare utilization due to uncertainty about hospital timing [3]. This may result in unnecessary interventions like early hospitalization, vaginal examinations, oxytocin use, amniotomy, prolonged stays, dystocia misdiagnosis, and unwarranted cesarean delivery [9]. Studies show women often struggle to differentiate between premonitory and active labor signs and complications requiring evaluation [9]. Healthcare providers help bridge this knowledge gap. Structured antenatal education has been shown to enhance maternal confidence and the ability to recognize labor signs. Research among Saudi first-time mothers demonstrated significant improvements in their knowledge and perceptions following participation in educational sessions [10]. Similarly, other studies have indicated that antenatal education helps reduce fear related to childbirth and promotes better preparedness for delivery [11].

Antenatal care (ANC) provides healthcare throughout pregnancy, including observation, education, and medical treatment [4]. ANC education informs pregnant women about pregnancy changes, fetal development, labor stages, warning signs, and pain relief techniques [12]. Women receiving continuous support from healthcare professionals (HCPs) during labor experience better birth outcomes, including lower emergency cesarean rates and higher childbirth satisfaction [4]. The World Health Organization (WHO) emphasizes that intrapartum care influences childbirth experience and recommends at least four facility visits during pregnancy [13]. In Saudi Arabia’s Vision 2030, ANC is vital to the safe birth system [14]. A study examining the impact of structured antenatal education on childbirth self-efficacy in Saudi Arabia found increased maternal confidence in the intervention group [8]. Studies have shown antenatal interventions positively affect postpartum maternal depression, suggesting the need for enhanced prenatal care and health education [15,16]. Research in Saudi Arabia revealed a knowledge gap among women regarding pregnancy concerns, highlighting the importance of targeted health education strategies and HCPs’ role [17]. A study in primary healthcare settings found a significant relationship between HCP guidance and women’s childbirth preparedness, enabling recognition of danger signs and quick decision-making [18]. Women’s preferences for maternity care professionals depend on being informed about care options and professionals understanding their specific needs [19,20].

Healthcare professionals significantly influence women’s sense of control during pregnancy and childbirth. When HCPs provide essential information, instructions, support decision-making, and respect preferences, it positively affects women’s sense of choice and control [21]. This impact relates to information provided during antenatal visits, where women adapt to childbirth pain through position changes and breathing exercises [22]. HCPs are crucial in shaping pregnant women’s understanding of pregnancy risks. Effective communication about risks helps women identify factors influencing risk perception, enhancing HCP-patient relationships and improving pregnancy outcomes [23]. When HCPs provide information, support, and respect preferences, it positively affects women’s sense of control [24]. During antenatal visits, HCP guidance enables women to adapt to childbirth pain through position changes and breathing exercises [25]. HCPs shape women’s understanding of pregnancy risks through effective communication, improving outcomes [26]. Previous studies have focused on HCP responsibilities and maternal education in Saudi Arabia’s healthcare system, with an emphasis on maternity and women’s health. However, no research has explored the relationship between HCPs’ educational role and Saudi pregnant women’s perception of active labor signs. This study highlights the need for collaboration among midwifery teams and healthcare providers to bridge clinical research gaps and enhance patient care.

### Significance of the study

Pregnancy and childbirth are transformative experiences that require adequate awareness and preparedness to ensure safe outcomes for both mother and child. One of the most critical challenges in maternal health is the limited knowledge among pregnant women about the active signs of labor. Failure to recognize these signs may result in delayed hospital admission, increasing the risks of maternal and neonatal complications such as prolonged labor, fetal distress, and emergency cesarean section [27]. Studies in Saudi Arabia indicate that women’s awareness of labor signs and complications remains insufficient. For instance, a study in Riyadh found that only 18.4% of pregnant women were aware of labor complications [28]. These findings highlight a substantial knowledge gap that can compromise timely decision-making during labor. The current study aims to improve maternal and neonatal outcomes by enabling timely hospital admission and reducing risks associated with delayed recognition of labor signs. It supports Saudi Vision 2030 health priorities, which emphasize women’s empowerment, health promotion, and quality maternal care, and highlight the importance of integrating structured, provider-led educational interventions into routine antenatal care programs. This study is the first to assess the knowledge level of pregnant Saudi women regarding signs of active labor and the role of healthcare providers.

### Theoretical framework: Health Belief Model

**Figure 1:**
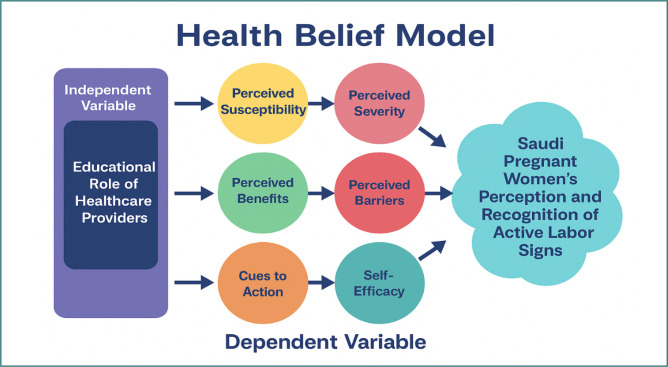
Conceptual framework based on the Health Belief Model (HBM), illustrating the relationship between the educational role of healthcare providers (independent variable) and Saudi pregnant women’s perception and recognition of active labor signs (dependent variable). The six HBM constructs—perceived susceptibility, perceived severity, perceived benefits, perceived barriers, cues to action, and self-efficacy—serve as mediating factors in this relationship.

To guide the conceptual foundation of this study, the Health Belief Model (HBM) was adopted (Figure 1). Initially developed by Becker [29], the HBM is a well-established psychological framework for explaining and predicting health-related behaviors by analyzing individuals’ beliefs and perceptions. Within the context of this research, the model provides a lens through which to examine how the educational role of healthcare providers (independent variable) influences Saudi pregnant women’s perception and recognition of active labor signs (dependent variable). The six central constructs of the HBM are mapped to this relationship as follows.

- **Perceived susceptibility** refers to a woman’s belief in her likelihood of experiencing complications if she fails to identify labor signs accurately. The educational role of healthcare providers enhances perceived susceptibility by highlighting personalized risks, such as maternal age, medical history, or prior pregnancy experiences. By recognizing their vulnerability, women become more attentive to bodily changes, thereby strengthening their perception and recognition of active labor signs.
- **Perceived severity** reflects a woman’s belief regarding the seriousness of potential consequences if labor signs are not recognized in a timely manner. Healthcare providers contribute to this construct by explaining the risks of delayed hospital admission, such as prolonged labor, fetal distress, or maternal complications. When women recognize the seriousness of these consequences, they are more likely to respond promptly to suspected signs of active labor.
- **Perceived benefits** relate to a woman’s belief in the positive outcomes associated with recognizing and acting upon active labor signs. Through structured antenatal education, providers emphasize that early recognition and timely hospital presentation support safer deliveries, reduce complications, and improve neonatal outcomes. By strengthening perceived benefits, healthcare providers motivate pregnant women to apply the knowledge they receive to real-life scenarios.
- **Perceived barriers** encompass cultural norms, misconceptions, emotional concerns, or logistical challenges that hinder the accurate recognition of labor signs. Healthcare providers address these barriers by identifying common myths, clarifying misconceptions, involving family members in counseling sessions, and offering solutions such as birth plans or transport arrangements. By reducing these obstacles, women are better able to interpret signs accurately and act without hesitation.
- **Cues to action** are external or internal triggers that stimulate readiness to engage in a health behavior. In this study, cues include reminders from healthcare providers, informational leaflets, mobile messages, partner education, and antenatal discussions. These cues serve as prompts that increase awareness and motivate pregnant women to apply their knowledge when signs of labor begin to appear.
- **Self-efficacy** refers to a woman’s confidence in her ability to correctly recognize and respond to labor signs. Provider-led education, which may include demonstrations, role-plays, and return demonstrations, significantly increases this confidence. As self-efficacy grows, women are more likely to correctly differentiate true labor from false labor and to make timely and appropriate decisions, such as contacting their provider or going to the hospital. Taken together, the HBM constructs explain how the educational role of healthcare providers strengthens women’s perceptions, reduces misconceptions, and increases readiness to act upon active labor signs. In this way, the HBM provides a comprehensive conceptual pathway linking the independent and dependent variables of this study [30].

### Aim of the study

This study aimed to evaluate how Saudi pregnant women’s knowledge, comprehension, and perception of the symptoms of active labor are impacted by the educational role of healthcare providers.

### Research objectives

- To assess the level of Saudi pregnant women’s knowledge and perception regarding the signs of active labor.
- To examine the extent and type of educational support provided by healthcare providers concerning active labor signs.
- To evaluate the relationship between healthcare providers’ educational role and pregnant women’s recognition of active labor signs.
- To identify perceived barriers and facilitators influencing pregnant women’s ability to recognize and respond to signs of active labor.
- To determine the predictive power of healthcare providers’ educational role (independent variable) on women’s perception and recognition of active labor signs (dependent variable).

### Research hypotheses

**H1:** Saudi pregnant women who receive structured education from healthcare providers demonstrate significantly higher knowledge of active labor signs compared to those who receive minimal or no structured education.

**H2:** There is a significant positive correlation between the educational role of healthcare providers and pregnant women’s perception of active labor signs.

**H3:** Higher self-efficacy (as influenced by healthcare provider education) is significantly associated with timely recognition and response to active labor signs.

**H4:** Perceived barriers (e.g., cultural norms, lack of family support, or emotional concerns) moderate the relationship between healthcare provider education and women’s recognition of active labor signs.

**H5:** Cues to action provided by healthcare providers (e.g., reminders, educational materials, counseling) significantly increase the likelihood of pregnant women accurately identifying and acting upon active labor signs.

## MATERIAL AND METHODS

### Research design

A descriptive cross-sectional correlational research design was used to assess the relationship between the health care provider’s educational role and Saudi pregnant women’s perceptions about signs of active labor in King Abdul-Aziz Medical City, Jeddah. The cross-sectional design involves collecting relevant information at a single point in time (during pregnancy). It helps identify patterns, trends, and potential relationships between variables, which can inform further research or interventions [31].

### Research setting

The study was conducted at the Obstetrics and Gynecology Outpatient Department (OPD) at King Abdul-Aziz Medical City (KAMC) in Jeddah. KAMC is a military tertiary hospital that operates several healthcare facilities. The hospital offers free emergency, inpatient, and outpatient care for eligible National Guard employees and their families. The outpatient department includes many clinics, each with sub-clinics that provide distinct services. Obstetrics and Gynecology clinics are among the main outpatient clinics, offering eight clinics available five days a week with morning and afternoon appointments. Each clinic is staffed by at least one Obstetrics and Gynecology Consultant, with varying levels of residents. The data were collected in the OPD waiting areas.

### Study population

Pregnant women attending the antenatal clinic at KAMC OPD were recruited for the current study. The inclusion criteria were: Saudi women who were pregnant and willing to participate in the current study.

### Exclusion criteria

Pregnant women attending the antenatal clinic with a known indication for planned elective cesarean section were excluded from the study. However, it is acknowledged that excluding this group may limit the external validity of the findings, as women scheduled for elective cesarean deliveries often still receive antenatal education and may possess relevant perceptions and knowledge about the signs of active labor. Future studies could consider including this subgroup to provide a more comprehensive understanding of labor-related awareness across all antenatal populations.

### Sampling and sampling techniques

RaoSoft software was employed to determine the necessary sample size for this research. Considering the estimated number of pregnant women visiting the Obstetrics and Gynecology Outpatient Department at King Abdulaziz Medical City, a minimum of 377 participants was calculated. This calculation was based on a medium effect size (0.30), an 80% statistical power, a 5% margin of error, and a 90% confidence level. The choice of a 90% confidence level was influenced by the exploratory nature of the study, resource limitations, and the uniformity of the target population, which permitted a slightly reduced confidence level without significantly affecting reliability. This strategy ensured a balance between statistical accuracy and practicality within the clinical environment.

A convenience sampling method, a form of nonprobability sampling, was used to select eligible Saudi pregnant women who met the inclusion criteria and were present in the outpatient department during the data collection period. This approach was suitable due to the ease of accessing participants in the clinical setting and the study’s descriptive-correlational design, which focused on exploring relationships rather than establishing population-wide causality [32].

### Data collection instrument

A structured questionnaire was used for data collection, adapted and developed from the instrument by Ijang *et al.* [33]. To ensure contextual suitability, the tool underwent cultural and linguistic adaptation for the Saudi population. The adaptation process included expert review, translation refinement, and pilot testing to ensure cultural relevance, conceptual clarity, and appropriateness of the items.

The questionnaire consisted of three main sections:

Section A: Collected participants’ socio-demographic and obstetric data, including age, education, residence, number of antenatal visits, gravidity, parity, and gestational age.

Section B: Assessed knowledge and perceptions regarding active labor signs. This section comprised eight items—six dichotomous items evaluating knowledge of labor signs (Yes = 1, No = 0; total score 0–6) and two multiple-choice items identifying sources of information and coping methods during contractions.

Section C: Explored women’s expectations of healthcare providers’ educational role. Ten items evaluated aspects such as communication effectiveness, adequacy of information, satisfaction, and preparedness for labor. Additional multiple-choice questions captured preferred educational methods and the specific topics discussed during antenatal visits.

### Validity and reliability

The questionnaire was translated into Arabic and then back-translated into English to ensure linguistic and conceptual accuracy. To strengthen cultural validity, three bilingual experts in obstetrics and maternal health evaluated the instrument for cultural relevance, clarity, and comprehensibility within the Saudi context. Minor modifications were made to align terminology and examples with local maternity practices and patient experiences. The content validity index (CVI) and expert agreement (κ = 0.98) confirmed strong content validity.

A pilot study involving 38 women (10% of the target sample) was conducted to assess the instrument’s clarity, acceptability, and reliability. Feedback from the pilot led to the rewording of two items to improve clarity and comprehension.

Internal consistency reliability was assessed using Cronbach’s alpha. The reliability coefficients were 0.60 for the knowledge scale and 0.73 for the healthcare provider educational role scale. While the alpha value for the knowledge scale was borderline, it was considered acceptable given the small number of dichotomous items (*n* = 6) and the exploratory nature of the study. Moreover, item-total correlations were reviewed to ensure adequate contribution of each item to the scale’s construct. Future research is recommended to further refine and expand the knowledge scale to enhance its internal consistency.

### Data collection process

Data were collected using a structured, self-administered Arabic-language questionnaire. At the beginning of each session, the researcher welcomed participants, introduced herself, explained the study objectives and procedures, and obtained informed consent in accordance with ethical standards. A total of 437 Saudi pregnant women were recruited, of whom 38 participated in a pilot study to evaluate the clarity, completeness, and reliability of the instrument. These participants were excluded from the main analysis, resulting in a final sample of 399 women. Data collection took place in the Obstetrics and Gynecology Clinic waiting area, located on the first floor of the ACC building, within the outpatient department. Participants completed the questionnaire while waiting for their scheduled appointments, which took approximately 7 to 10 minutes. Conducting data collection in the waiting area was considered practical and time-efficient, as it allowed access to a large number of eligible participants without disrupting clinical workflows. To minimize potential distractions or peer influence, participants were seated separately and instructed to complete the questionnaire individually. Although the self-administered format may limit the depth of responses for perception-related items, it was selected to encourage privacy, autonomy, and honest disclosure, particularly on sensitive or experiential questions. To enhance accuracy, the researcher was available nearby to clarify any ambiguous items or respond to participant queries. Future studies may consider an interviewer-administered approach or a mixed-methods design to capture more nuanced insights into women’s understanding and perceptions of labor signs.

### Data management and analysis

Data were analyzed using the Statistical Package for the Social Sciences (SPSS), version 29. Descriptive statistics, including frequencies, percentages, means, standard deviations, and distributions, were used to summarize participants’ demographic and study variables. Inferential statistics, specifically the Chi-square test, were employed to examine associations between women’s perceptions of active labor signs and the study variables. A *P* value ≤ 0.05 was considered statistically significant for hypothesis testing.

**Table 1:** Distribution of participants according to demographic characteristics, *n* = 399

Variable	Category	*n* (%) / Mean ± SD
**Age (years)**	18–25	39 (9.8)
	26–35	229 (57.4)
	36–45	131 (32.8)
**Educational level**	Primary	9 (2.2)
	Intermediate	12 (3.0)
	Secondary	78 (19.5)
	University	258 (64.7)
	Others	42 (10.5)
**Residence**	Jeddah	201 (50.4)
	Mecca	144 (36.1)
	Other	54 (13.5)
**Pregnancy clinical visits**	<4	77 (19.3)
	≥4	322 (80.7)
**Gestational age (weeks)**	<28	58 (14.6)
	28–30	26 (6.5)
	31–35	64 (16.0)
	36–40	251 (62.9)
**Number of pregnancies**	Mean ± SD	3.6 ± 2.2
	1–3	226 (56.6)
	4–6	136 (34.1)
	≥7	37 (9.3)
**Number of deliveries**	Mean ± SD	2.2 ± 1.8
	0	68 (17.0)
	1–3	244 (61.2)
	4–6	78 (19.5)
	≥7	9 (2.3)
**Number of abortions**	Mean ± SD	0.5 ± 0.8
	0	266 (66.7)
	1	90 (22.6)
	≥2	43 (10.7)
**Number of preterm labors**	Mean ± SD	0.15 ± 0.5
	0	356 (89.2)
	1	32 (8.0)
	≥2	11 (2.8)

## RESULTS

Table 1 presents the distribution of the study participants (*n* = 399) by demographic characteristics. The majority of participants (57.4%) were aged 26–35 years, while 32.8% were aged 36–45 years. More than half of the participants (64.7%) held a university degree, and 19.5% had completed secondary education. Approximately half of the participants resided in Jeddah, and 36% were from Makkah.

**Figure 2:**
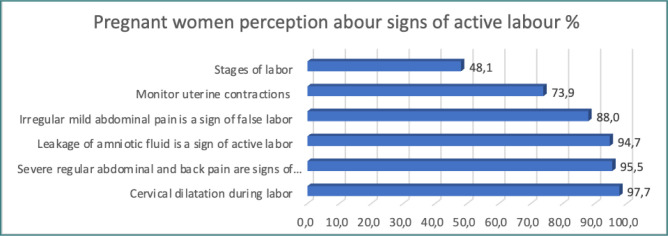
Pregnant women’s perception about signs of active labor

**Figure 3:**
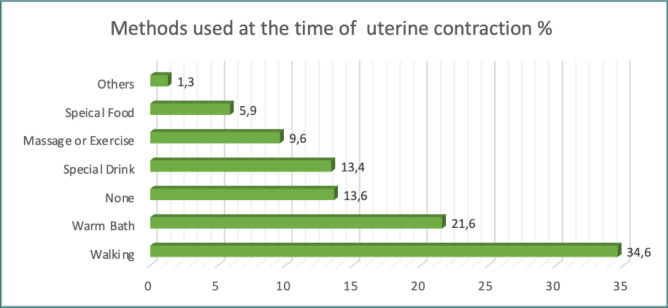
Methods used at the time of the contraction

**Figure 4:**
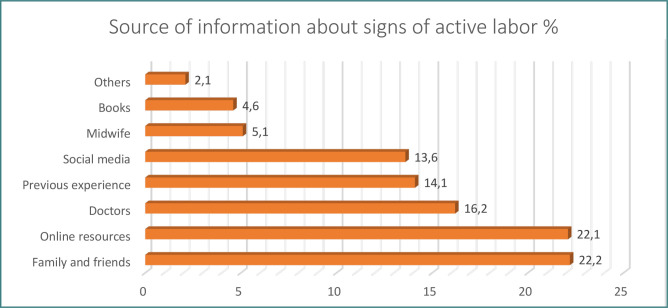
Sources of information about signs of active labor

A total of 80.7% of participants reported having four or more antenatal visits during their current pregnancy, and 62.9% had completed 36–40 weeks of gestation. More than half (56.6%) had experienced one to three previous pregnancies, while about 9% reported more than seven pregnancies. The mean gravidity was 3.6 ± 2.2. In terms of parity, 61.2% had one to three previous deliveries, whereas 17% had not delivered before; the mean parity was 2.2 ± 1.8. The mean number of abortions was 0.5 ± 0.8. Regarding preterm labor, 8% had experienced one preterm birth, and 11 participants reported two or more preterm labors.

Figure 2 shows pregnant women’s perception of signs of active labor. 97.7% of women were aware of gradual cervical dilatation during active labor, and 95.5% knew that severe regular abdominal and back pain are signs of active labor. Moreover, the results showed that less than half of the women were aware of the stages of labor.

Participants reported that walking was the most commonly used method during uterine contractions (34.6%), followed by taking a warm bath (21.6%). In addition, 13.6% of women reported not using any method during uterine contractions (Figure 3).

Figure 4 illustrates the sources of information women use to recognize signs of active labor. Family and friends (22.2%) and online resources (22.1%) were the most commonly reported sources. The least used sources were consulting midwives and books.

Table 2 illustrates the educational role of healthcare providers among the 399 women included in the study. The majority of participants (96%) reported feeling comfortable asking healthcare providers questions about the signs of active labor. Approximately 71.9% of participants indicated that communication with healthcare providers regarding these signs was effective or very effective. In addition, 69.9% of the women reported feeling confident or very confident in identifying the signs of active labor. Furthermore, 74.2% of participants rated the quality of information provided by healthcare providers about active labor signs as sufficient or very sufficient. Overall, 77% of women were satisfied or very satisfied with the educational methods used by healthcare providers. Most participants (86%) reported being prepared or very prepared to identify the signs of active labor, and an equal proportion (86%) stated that healthcare providers adequately prepared them for the active stage of normal labor.

**Table 2:** Participant responses on perceptions of healthcare providers’ educational role, *n* = 399

Item	1	2	3	4	5
**Communicate effectively with health care providers**	4 (1.0%)	8 (2.0%)	100 (25.1%)	159 (39.8%)	128 (32.1%)
**Confidence in recognizing labor signs**	5 (1.2%)	15 (3.8%)	116 (29.1%)	150 (37.6%)	113 (28.3%)
**Quality of information provided**	2 (0.5%)	12 (3.0%)	89 (22.3%)	187 (46.9%)	109 (27.3%)
**Satisfaction with educational methods**	2 (0.5%)	10 (2.5%)	82 (20.5%)	195 (48.9%)	110 (27.6%)
**Preparedness to identify labor signs**	2 (0.5%)	8 (2.0%)	46 (11.5%)	170 (42.6%)	173 (43.4%)
**Item**				Yes	No
**Comfortable asking provider questions about labor signs**				383 (96.0%)	16 (4.0%)
**Providers adequately prepared for active stage of labor**				383 (96.0%)	58 (14.5%)

Likert scale reference (Left to Right): 1 = Very ineffective/Very unconfident/Very insufficient/Very unsatisfied/Very unprepared 2 = Ineffective/Unconfident/Insufficient/Unsatisfied/Unprepared 3 = Natural 4 = Effective/Confident/Sufficient/Satisfied/Prepared 5 = Very effective/Very confident/Very sufficient/Very satisfied/Very prepared

**Figure 5:**
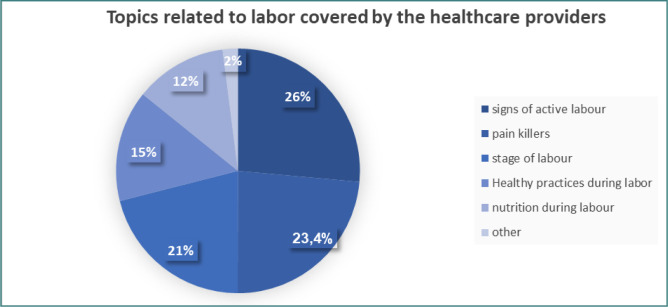
Educational topics covered by HCPs

**Figure 6:**
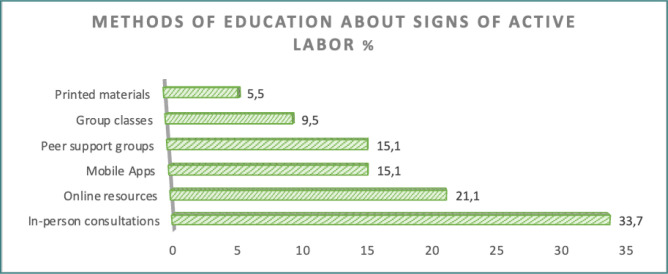
Participant opinions regarding the most effective method for education the signs of active labor

**Table 3:** The relationship between the demographic profile of participants (low, high) and the perception of active signs of labor, *n* = 399

Variable	Low	High	χ^2^	*P* value
**Age**			5.22	0.074
18-25	6 (15.4)	33 (84.6)		
26-35	27 (11.8)	202 (88.2)		
36-45	7 (5.4)	124 (96.6)		
**Educational level**			2.42	0.489
Less than secondary	3 (14.3)	18 (85.7)		
Secondary	10 (12.8)	68 (87.2)		
University	25 (9.7)	233 (90.3)		
Others	2 (4.8)	40 (95.2)		
**Residence**			4.19	0.122
Jeddah	5 (9.3)	49 (90.7)		
Mecca	26 (12.9)	175 (87.1)		
Other	9 (6.3)	135 (93.7)		
**Pregnancy clinical visits**			1.92	0.166
Less than 4	11 (14.3)	66 (85.7)		
4 and above	29 (9.0)	293 (91.0)		
**Gestational age**			15.87	0.002*
36-40 weeks	17 (6.8)	234 (93.2)		
31-35 weeks	15 (23.4)	49 (76.6)		
28-30 weeks	2 (7.7)	24 (93.3)		
Less than 28 weeks	6 (10.3)	52 (89.7)		
**Number of pregnancies**			6.11	0.047*
1-3	30 (13.3)	196 (86.7)		
4-6	8 (5.9)	128 (94.1)		
7+	2 (5.4)	35 (94.6)		
**Number of deliveries**			22.36	0.001*
0	17 (25.0)	51 (75.0)		
1-3	20 (8.2)	224 (91.8)		
4-6	2 (2.6)	76 (97.4)		
7+	1 (11.1)	8 (89.9)		
**Number of abortions**			3.73	0.154
0	31 (11.7)	235 (88.3)		
1	8 (8.9)	82 (91.1)		
2+	1 (2.3)	42 (97.7)		
**Number of preterm labors**			1.89	0.387
0	38 (16.7)	318 (89.3)		
1	2 (6.3)	30 (93.7)		
2+	0 (0)	11 (100)		

*Significant association

Figure 5 presents the labor-related topics covered by healthcare providers. The most frequently discussed topic was the signs of active labor (26.2%), followed by the use of painkillers (23.4%) and nutrition during labor (12.1%).

Figure 6 displays participants’ opinions regarding the most effective methods for education about the signs of active labor. In-person consultation with family and friends who are not in the medical field was the most reported method (33.7%), followed by online resources (21.1%). Printed materials were the least preferred source (5.5%).

Table 3 presents the overall perception of signs of active labor, categorized as low or high, and its association with participants’ demographic variables, using the Chi-square test. The results revealed a statistically significant association between participants’ perception of the signs of active labor and their gestational age, number of pregnancies, and number of deliveries (*P* < 0.05).

Women at 36–40 and 28–30 weeks of gestation were more likely to correctly recognize the signs of active labor. In addition, women with more pregnancies and deliveries reported a higher level of perception than those with fewer.

Table 4 presents the association between healthcare providers’ educational role and women’s perception of signs of active labor. The Chi-square test revealed that the significant factors associated with women’s perception were feeling comfortable asking healthcare providers questions, self-confidence in recognizing the signs of active labor, and adequate preparation to identify these signs (*P* < 0.05).

**Table 4:** The relationship between the health care provider’s educational role (low, high) and the women’s perception about active signs of labor, *n* = 399

Variable	Low	High	χ^2^	*P* value
**Do you feel comfortable asking your healthcare provider questions about signs of active labor?**			4.14	0.042*
Yes	36 (9.4)	347 (90.6)		
No	4 (25.0)	12 (75.0)		
**How effective is your communication with the health care provider about signs of active labor**			1.32	0.517
Effective	26 (9.1)	261 (90.1)		
Natural	12 (12.0)	88 (88.0)		
Ineffective	2 (16.7)	10 (83.3)		
**How confident are you in your ability to recognize the signs of active labor?**			19.48	0.001*
Confident	15 (5.7)	248 (94.3)		
Natural	19 (16.4)	97 (83.6)		
Unconfident	6 (30.0)	14 (70.0)		
**How would you rate the quality of information provided by your healthcare provider about the signs of active labor?**			4.69	0.095
Sufficient	24 (8.1)	272 (91.9)		
Natural	14 (15.7)	75 (84.3)		
Insufficient	2 (14.3)	12 (85.7)		
**How satisfied are you with the educational methods used by your healthcare provider?**			1.26	0.533
Satisfied	28 (9.2)	277 (90.8)		
Natural	10 (12.2)	72 (87.8)		
Unsatisfied	2 (16.7)	10 (83.3)		
**How prepared do you feel to identify the signs of active labor?**			8.12	0.017*
Prepared	29 (8.5)	314 (91.5)		
Natural	8 (17.4)	38 (82.6)		
Unprepared	3 (30.0)	7 (70.0)		
**Do you feel that your healthcare provider has adequately prepared you for identifying active labor?**			2.27	0.132
Yes	31 (9.1)	310 (90.9)		
No	9 (15.5)	49 (84.5)		

**Table 5:** Multivariate binary logistic regression of perception (low-high) factors

Variable	OR (95% CI)	*P* value
**Age**		
18–25 (Ref)	-	-
26–35	0.74 (0.24-2.24)	0.592
36–45	1.39 (0.35-5.54)	0.640
**Educational level**		
Secondary and less (Ref)	-	-
University	1.27 (0.62-2.61)	0.516
**Residence**		
Jeddah	0.66 (0.24-1.79)	0.411
Mecca	1.35 (0.44-4.07)	0.600
Other (Ref)	-	-
**Pregnancy clinical visits**		
Less than 4 (Ref)	-	-
4 and above	2.04 (1.06-4.07)	0.033
**Gestational age**		
36-40 weeks	4.15 (1.68-9.93)	0.002
31-35 weeks	1.25 (0.25-6.14)	0.785
28-30 weeks	0.76 (0.24-1.79)	0.607
Less than 28 weeks (Ref)	-	-
**Number of pregnancies**		
1-3 (Ref)	-	-
4-6	0.47 (0.15-1.52)	0.208
7+	3.35 (0.87-7.23)	0.064
**Number of deliveries**		
0 (Ref)	-	-
1-3	5.41 (2.21-13.3)	0.001
4+	6.54 (2.41-14.2)	0.006
**Number of abortions**		
0 (Ref)	-	-
1	0.82 (0.31-2.19)	0.693
2+	3.32 (0.87-7.23)	0.101
**Number of preterm labors**		
0 (Ref)	-	-
1	1.51 (0.31-4.23)	0.608
2+	1.22 (0.45-4.01)	0.982

Table 5 shows that the logistic regression model explained approximately 25.3% of the variance in perception levels (Nagelkerke R^2^ = 0.253), indicating a moderate explanatory power. The analysis identified significant predictors influencing perception, including the number of pregnancy clinical visits, gestational age, and number of deliveries. Women who attended four or more antenatal visits were twice as likely to have a high perception level as those with fewer visits (OR = 2.04; *P* = 0.033), highlighting the role of regular follow-up in enhancing awareness and understanding. Similarly, those in late pregnancy (36–40 weeks) were about four times more likely to demonstrate high perception (OR = = 4.15; *P* = 0.002), possibly due to increased education and exposure as pregnancy progresses. Furthermore, multiparous women showed significantly higher perception levels than primigravida women, with odds ratios of 5.41 for 1–3 deliveries (*P* = 0.001) and 6.54 for 4 or more deliveries (*P* = 0.006), suggesting that experience from previous childbirths strengthens understanding and perception. A near-significant trend was observed for women with seven or more pregnancies (OR = 3.35, *P* = 0.064), indicating a potential positive association between higher parity and perception. In contrast, demographic factors such as age, educational level, residence, number of abortions, and preterm labor did not significantly affect perception levels (*P* > 0.05), suggesting that clinical exposure and maternal experience are more influential determinants of perception than sociodemographic characteristics.

## DISCUSSION

The present study revealed that 95.5% of participants correctly identified severe, regular abdominal and back pain as key indicators of active labor. While this demonstrates a high level of awareness, it predominantly reflects recognition of the most obvious labor symptoms. Awareness of less apparent signs—such as bloody show or rupture of membranes—was comparatively lower, indicating gaps in comprehensive understanding. This finding suggests that while foundational knowledge is strong among Saudi women, antenatal education should extend beyond pain recognition to encompass a broader spectrum of labor indicators, fostering more informed self-monitoring and timely decision-making.

The study also highlighted that family, friends, and online platforms were frequently cited as sources of information, with over 22% of participants relying on informal channels. This contrasts with studies from other settings, such as Vogels-Broeke *et al*. [34], where professional guidance predominates. In Saudi Arabia, this reliance on informal sources may be influenced not only by accessibility but also by cultural norms that prioritize family advice and social networks, as well as systemic healthcare factors, including limited consultation time and variability in antenatal education coverage. Addressing these barriers by integrating structured, culturally sensitive, and digitally accessible educational interventions can help ensure that women receive reliable information to make informed decisions.

Furthermore, significant associations were observed between women’s perception of active labor signs and factors such as gestational age, parity, and previous deliveries. These results contrast with Félix *et al*. [9], who found no correlation between maternal characteristics and knowledge. However, similar associations were reported in previous studies such as Wassihun *et al*. [35] and Hailu *et al*. [36], supporting the idea that maternal awareness may be contextually influenced by local healthcare practices, exposure to structured education, and personal experiences. This discrepancy suggests that maternal awareness may be contextually influenced by local healthcare practices, exposure to structured education, and personal experiences. It underscores the importance of considering sociocultural and systemic factors when designing antenatal educational programs.

The study emphasizes the pivotal role of healthcare providers in enhancing maternal knowledge. Women who received structured provider-led education reported higher confidence in recognizing labor signs and greater preparedness for childbirth. These findings align with research by Bohren *et al*. [37], Dibazari *et al*. [38], Moinuddin *et al*. [39], and Said *et al*. [40], who demonstrated that structured antenatal education improves awareness, reduces medical interventions, and enhances psychological readiness. Similarly, Alharbi *et al*. [41] and Bhavana *et al*. [42] found that targeted education increases women’s understanding of normal and abnormal pregnancy signs. The WHO also emphasizes comprehensive antenatal education, advocating for both physical preparation and emotional readiness [13]. Globally, women’s experiences highlight the need for clear and respectful communication during antenatal care, as evidenced by Oladapo *et al*. [43] and Sidani Yusuf & Feghali [44]. In the Saudi context, the predominance of informal information sources highlights the urgent need to strengthen structured, evidence-based educational programs. Strengthening digital health solutions is critical, particularly when evidence indicates that education level predicts knowledge of pregnancy danger signs, as shown in Miyauchi *et al*. [45]. Integrating reliable digital platforms alongside face-to-face provider education can enhance accessibility, improve patient-provider communication, and empower women to make safe, informed decisions during labor.

While this study offers significant insights, certain limitations should be acknowledged. The dependence on self-reported data could lead to social desirability bias. Additionally, the cross-sectional design restricts causal inferences, and the omission of women scheduled for elective cesarean sections may diminish the generalizability of the findings to the wider pregnant population. Previous literature, such as Wulandari and Laksono [46] and Baeschlin & Mueller [47], further highlights the complexity of assessing labor knowledge and early labor signs, pointing to the need for robust, multi-method research approaches. Future studies should investigate longitudinal outcomes of antenatal education interventions and analyze how cultural and systemic factors influence information-seeking behaviors.

The current study explored factors influencing Saudi pregnant women’s perception of active labor signs, with a particular focus on the educational role of healthcare providers. The logistic regression analysis identified three key predictors—number of antenatal visits, gestational age, and parity—that significantly influenced perception levels, collectively accounting for approximately 25.3% of the variance (Nagelkerke R^2^ = 0.253). These results highlight the pivotal role of maternal experience and engagement with antenatal care in shaping labor-related awareness.

Women who attended four or more antenatal visits were twice as likely to demonstrate high levels of perception (OR = 2.04,*P* = 0.033), underscoring the educational impact of consistent prenatal care. Regular interactions with healthcare professionals provide repeated opportunities for counseling, clarification of doubts, and reinforcement of essential maternal health information. This finding aligns with a study by Alharbi *et al*. [41] which reported that frequent antenatal attendance significantly enhances maternal knowledge regarding labor preparedness. Similarly, another study attributed improved awareness of birth preparedness and danger signs to structured education delivered by midwives and nurses during routine visit [43].

Perception levels were notably higher among women in late pregnancy (36–40 weeks), with an odds ratio of 4.15 (P = 0.002). This trend likely reflects the cumulative effect of prenatal education and increased psychological readiness as delivery nears. As gestation progresses, women receive more targeted counseling on labor onset and appropriate responses, which enhances knowledge retention and its practical application. These findings are consistent with Alzahrani *et al*. [41] who observed that third-trimester women exhibited greater recognition of labor symptoms. However, Oladapo *et al*. [43] noted persistent knowledge gaps despite advancing gestational age, suggesting that the effectiveness of education may depend on delivery methods and provider engagement.

Multiparous women showed significantly higher perception levels than primigravidas, with odds ratios of 5.41 for 1–3 deliveries and 6.54 for 4 or more deliveries. This strong association indicates that experiential learning from previous pregnancies enhances understanding of labor signs. Studies by Yusuf *et al*. [44] support this, demonstrating that multiparity positively influences recognition of labor onset and appropriate behavioral responses. Prior childbirth experiences help women distinguish between true labor and false alarms, fostering confidence and preparedness. Contrastingly, other studies found no significant difference between primigravida and multigravida women, suggesting that well-structured antenatal education can bridge experiential gaps when effectively implemented [45].

Interestingly, variables such as age, education level, residence, abortion history, and preterm labor did not significantly predict perception levels. This suggests that clinical exposure and maternal experience are more influential than sociodemographic background in shaping labor knowledge. These findings are consistent with a research by Moinuddin *et al*. [39], who reported that standardized educational support minimized the impact of demographic differences on maternal health awareness. However, Wulandari & Laksono [46] identified education and urban residence as significant determinants of maternal knowledge, pointing to potential contextual variations in health literacy and access to care.

The current study findings emphasize the critical role of healthcare providers in empowering pregnant women through continuous, experience-based, and gestation-tailored education. Regular antenatal visits serve not only as clinical checkups but also as educational encounters that strengthen women’s preparedness for childbirth. The significant contribution of parity and late gestation reinforces the importance of integrating practical learning and individualized counseling into maternal care protocols. The absence of significant demographic influences suggests that effective provider–client communication and educational consistency can overcome traditional barriers related to age or formal education.

### Implications for clinical practice

The findings of this study underscore the critical role of healthcare providers in delivering effective antenatal education. Women reported feeling comfortable and well-prepared when communicating with their providers, suggesting that interpersonal interactions are essential for meaningful educational experiences. For clinical practice, this highlights the need for healthcare institutions to implement training programs that enhance providers’ communication skills, particularly in culturally sensitive settings such as Saudi Arabia, where cultural norms may influence patient-provider dynamics.

Empowering midwives to take an active role in educating pregnant women about the signs of active labor may foster more open dialogue, especially among primigravida women who may be less confident in seeking information. Encouraging such interactions can lead to deeper understanding and better preparedness.

Given that gestational age and parity were significantly associated with women’s perception of labor signs, healthcare providers should consider offering personalized antenatal education sessions. Tailoring content to meet the distinct needs of primigravida versus multiparous women may enhance the effectiveness of these programs. One of the key knowledge gaps identified in this study was a limited understanding of labor stages, which could be more effectively addressed through individualized educational strategies.

### Limitations

Despite offering valuable insights, several limitations must be considered:

The cross-sectional nature of the study prevents causal inference, and the sample consisted primarily of urban women attending antenatal clinics, limiting generalizability to rural or low socioeconomic groups. Data were gathered through self-administered questionnaires in waiting areas, potentially introducing social desirability or acquiescence bias.

The knowledge scale exhibited borderline reliability (Cronbach’s alpha = 0.60), indicating limited internal consistency. Dichotomous or multiple-choice questions may oversimplify perceptions or fail to capture a nuanced understanding. Dependence on self-reporting may lead to an overestimation of participants’ actual knowledge or preparedness. In addition, the research was conducted at a limited number of clinics within a single region, which may not accurately reflect the broader population of Saudi women.

### Recommendations

1. Design antenatal education initiatives that are tailored to various demographic groups, with specific content addressing the unique needs of primigravida and multiparous women.
2. Promote family involvement and encourage family members—particularly husbands—to participate in antenatal education sessions.
3. Update prenatal education methods by integrating innovative technologies, such as mobile applications and digital platforms, to enhance accessibility and engagement, and improve the overall quality of maternal care.
4. Future studies should adopt longitudinal designs to assess the sustained impact of antenatal education on maternal knowledge, behavior, and labor outcomes. This approach will provide deeper insights into the long-term effectiveness of educational interventions. Expanding future research to include more diverse groups would enhance the generalizability of the findings.

## CONCLUSION

This study underscores the pivotal role of healthcare providers in delivering effective antenatal education, emphasizing that its success is closely tied to the quality of provider-patient communication, the educational strategies employed, and the level of preparedness among Saudi pregnant women. Prior maternal experiences also contribute significantly to how educational messages are received and internalized. Antenatal education emerged as a vital component in preparing women for childbirth, equipping them with the knowledge and confidence needed to navigate labor effectively. The study identified key factors influencing women’s perception of active labor signs, including their comfort in engaging with healthcare providers, self-confidence in recognizing labor indicators, and overall preparedness to identify these signs. These findings highlight the importance of personalized, culturally sensitive, and interactive educational approaches to enhance maternal awareness and promote safe, timely decision-making during labor.

## Personal thanks

The researcher gratefully acknowledges the women who participated in this study for their valuable time, experiences, and insights, which were essential in advancing knowledge on maternal health and the role of antenatal education in recognizing signs of active labor. Appreciation is also extended to King Abdulaziz Medical City, Western Region, and the King Abdullah International Medical Research Center (KAIMRC), Jeddah, Saudi Arabia, for their support and ethical approval, as well as to the administrative staff of the Obstetrics and Gynecology Outpatient Department for their assistance during data collection.
